# Case-control study for colorectal cancer genetic susceptibility in EPICOLON: previously identified variants and mucins

**DOI:** 10.1186/1471-2407-11-339

**Published:** 2011-08-05

**Authors:** Anna Abulí, Ceres Fernández-Rozadilla, Virginia Alonso-Espinaco, Jenifer Muñoz, Victoria Gonzalo, Xavier Bessa, Dolors González, Joan Clofent, Joaquin Cubiella, Juan D Morillas, Joaquim Rigau, Mercedes Latorre, Fernando Fernández-Bañares, Elena Peña, Sabino Riestra, Artemio Payá, Rodrigo Jover, Rosa M Xicola, Xavier Llor, Luis Carvajal-Carmona, Cristina M Villanueva, Victor Moreno, Josep M Piqué, Angel Carracedo, Antoni Castells, Montserrat Andreu, Clara Ruiz-Ponte, Sergi Castellví-Bel

**Affiliations:** 1 Department of Gastroenterology, IDIBAPS, Hospital Clínic, CIBERehd, University of Barcelona, Barcelona, Catalonia, Spain; 2 Gastroenterology Department, Parc de Salut Mar, Institut Municipal d'Investigació Mèdica (IMIM), Pompeu Fabra University, Barcelona, Catalonia, Spain; 3 Galician Public Foundation of Genomic Medicine, CIBERER, Genomics Medicine Group, Hospital Clínico, Santiago de Compostela, University of Santiago de Compostela, Galicia, Spain; 4 Hospital Sant Pau, Barcelona, Catalonia, Spain; 5 Department of Gastroenterology, Hospital Meixoeiro, Vigo, Spain; 6 Department of Gastroenterology, Hospital de Ourense, Galicia, Spain; 7 Department of Gastroenterology, Hospital 12 de Octubre, Madrid, Spain; 8 Department of Medicine, Hospital General de Granollers, Barcelona, Catalonia, Spain; 9 Department of Gastroenterology, Hospital General Universitario de Valencia, Valencia, Spain; 10 Department of Gastroenterology, Hospital Mutua de Terrassa, Barcelona, Catalonia, Spain; 11 Department of Gastroenterology, Hospital Royo Villanova, Zaragoza, Spain; 12 Department of Gastroenterology, Hospital Central de Asturias, Oviedo, Asturias, Spain; 13 Department of Pathology and Gastroenterology, Hospital General d'Alacant, Alicante, Spain; 14 Section of Digestive Diseases and Nutrition and Cancer Center, University of Illinois at Chicago, IL 60612, USA; 15 Wellcome Trust Centre for Human Genetics, University of Oxford, Roosevelt Drive, Oxford OX3 7BN, UK; 16 Centre for Research in Environmental Epidemiology (CREAL), IMIM (Hospital del Mar Research Institute). CIBER Epidemiología y Salud Pública (CIBERESP), Barcelona, Spain; 17 IDIBELL-Institut Català d'Oncologia (ICO), CIBER Epidemiología y Salud Pública (CIBERESP), University of Barcelona, L'Hospitalet de Llobregat, Barcelona, Spain; 18 All authors are listed in the Acknowledgements section

**Keywords:** Colorectal Neoplasms, Genetic Predisposition to Disease, Single Nucleotide Polymorphism, Mucins, Genetic Association Studies

## Abstract

**Background:**

Colorectal cancer (CRC) is the second leading cause of cancer death in developed countries. Familial aggregation in CRC is also important outside syndromic forms and, in this case, a polygenic model with several common low-penetrance alleles contributing to CRC genetic predisposition could be hypothesized. Mucins and GALNTs (N-acetylgalactosaminyltransferase) are interesting candidates for CRC genetic susceptibility and have not been previously evaluated. We present results for ten genetic variants linked to CRC risk in previous studies (previously identified category) and 18 selected variants from the mucin gene family in a case-control association study from the Spanish EPICOLON consortium.

**Methods:**

CRC cases and matched controls were from EPICOLON, a prospective, multicenter, nationwide Spanish initiative, comprised of two independent stages. Stage 1 corresponded to 515 CRC cases and 515 controls, whereas stage 2 consisted of 901 CRC cases and 909 controls. Also, an independent cohort of 549 CRC cases and 599 controls outside EPICOLON was available for additional replication. Genotyping was performed for ten previously identified SNPs in *ADH1C*, *APC*, *CCDN1*, *IL6*, *IL8*, *IRS1*, *MTHFR*, *PPARG*, *VDR *and *ARL11*, and 18 selected variants in the mucin gene family.

**Results:**

None of the 28 SNPs analyzed in our study was found to be associated with CRC risk. Although four SNPs were significant with a *P*-value < 0.05 in EPICOLON stage 1 [rs698 in *ADH1C *(OR = 1.63, 95% CI = 1.06-2.50, *P*-value = 0.02, recessive), rs1800795 in *IL6 *(OR = 1.62, 95% CI = 1.10-2.37, *P*-value = 0.01, recessive), rs3803185 in *ARL11 *(OR = 1.58, 95% CI = 1.17-2.15, *P*-value = 0.007, codominant), and rs2102302 in *GALNTL2 *(OR = 1.20, 95% CI = 1.00-1.44, *P*-value = 0.04, log-additive 0, 1, 2 alleles], only rs3803185 achieved statistical significance in EPICOLON stage 2 (OR = 1.34, 95% CI = 1.06-1.69, *P*-value = 0.01, recessive). In the joint analysis for both stages, results were only significant for rs3803185 (OR = 1.12, 95% CI = 1.00-1.25, *P*-value = 0.04, log-additive 0, 1, 2 alleles) and borderline significant for rs698 and rs2102302. The rs3803185 variant was not significantly associated with CRC risk in an external cohort (MCC-Spain), but it still showed some borderline significance in the pooled analysis of both cohorts (OR = 1.08, 95% CI = 0.98-1.18, *P*-value = 0.09, log-additive 0, 1, 2 alleles).

**Conclusions:**

*ARL11*, *ADH1C*, *GALNTL2 *and *IL6 *genetic variants may have an effect on CRC risk. Further validation and meta-analyses should be undertaken in larger CRC studies.

## Background

Colorectal cancer (CRC) is the second leading cause of cancer death in developed countries [1]. Familial adenomatous polyposis and Lynch syndrome are the most frequent hereditary CRC syndromes with a more aggressive presentation, earlier onset and strong familial aggregation. However, they only correspond to a minority of the total CRC burden (~5%). Most genetic components involved in these less frequent hereditary forms were successfully identified in the past two decades and they correspond to rare, highly penetrant alleles that predispose to CRC. Genetic association analyses have been the strategy to identify predisposing CRC alleles in the last decade, firstly by studying a small number of such variants or single nucleotide polymorphisms (SNP) using candidate-gene approaches [2], and lately with an unbiased strategy by genome-wide association studies (GWAS) [3,4].

From the EPICOLON consortium [5], several genetic association candidate-gene efforts have been pursued since 2005 aiming to identify genetic susceptibility variants for CRC. In this manner, SNPs/genes were selected to be studied from the previously identified category (variants linked to CRC risk in previous studies), from human syntenic CRC susceptibility regions identified in mouse, from the CRC carcinogenesis-related pathways Wnt and BMP, from regions 9q22 and 3q22 with positive linkage in CRC families, and from the mucin gene family [6-8]

SNPs in genes selected in the previously identified category (*ADH1C*, *APC*, *CCDN1*, *IL6*, *IL8*, *IRS1*, *MTHFR*, *PPARG*, *VDR *and *ARL11*) have been analyzed in previous independent genetic association studies and they are *a priori *attractive candidates for genetic susceptibility to CRC [6,9-24]. The expression of most genes in this category are altered in CRC and they appear to be involved in important processes for CRC risk such as hereditary CRC (*APC*), alcohol metabolism (*ADH1C*), inflammation (*IL6*, *IL8*), cell cycle regulation (*CCDN1*), energy balance (*IRS1*, *PPARG*), methylation (*MTHFR*), vitamin D (*VDR*), and the RAS superfamily (*ARL11*). On the other hand, mucins are protein constituents of the mucous barrier that protects human epithelia from adverse conditions, and they are highly glycosylated by GALNT proteins. Mucins and GALNTs, members of the mucin gene family, can be found deregulated in CRC and other neoplasms and, although also interesting candidates, they have not been previously evaluated for CRC genetic susceptibility [25].

Here, we report results from a case-control association study for CRC risk in the EPICOLON cohort for previously identified SNPs in *ADH1C*, *APC*, *CCDN1*, *IL6*, *IL8*, *IRS1*, *MTHFR*, *PPARG*, *VDR *and *ARL11*, and selected variants within the mucin gene family.

## Methods

### Study populations

Studied subjects were mainly from EPICOLON, a prospective, multicenter, nationwide Spanish initiative http://www.aegastro.es/aeg/ctl_servlet?_f=16&grupo=4[5], comprised of two stages (2000-2001 and 2006-2008), where CRC cases and matched healthy controls were collected. DNA samples were extracted as previously described [6,7]. EPICOLON stage 1 corresponded to 515 CRC cases and 515 controls, whereas EPICOLON stage 2 consisted of 901 CRC cases and 909 controls. Additionally, an independent cohort of 549 CRC cases and 599 controls (MCC-Spain; http://www.creal.cat) was available for further replication of putative positive hits in EPICOLON. Cases and controls were matched for sex and age (± 5 years) and controls were negative for personal and family cancer history. All samples were obtained with informed consent reviewed by the ethical board of the corresponding hospital.

### Gene and SNP selection

Selected SNPs were included in the previously identified category if they corresponded to genetic variants linked to CRC risk by previously published independent studies, or in the mucin gene family if located on genes encoding for mucins and GALNT proteins. Mucins are protein constituents of the mucous barrier highly glycosylated by GALNT proteins. One relevant previously identified SNP was studied in each of the following genes: *ADH1C*, *APC*, *CCDN1*, *IL6*, *IL8*, *IRS1*, *MTHFR*, *PPARG*, *VDR *and *ARL11*. For the mucin gene family, 1-2 SNPs were selected from each of the following genes: *MUC7*, *MUC12*, *MUC13*, *MUC15*, *MUC16*, *MUC17*, *MUC19*, *MUC21*, *GALNT1*, *GALNTL2*, *GALNT10*, *GALNT14*, *GALNT4 *and *OVGP1*. Mucin gene selection did not intentionally include *GALTN12*. This gene is located on the 9q22 region and it was studied in our candidate-gene approach for regions with positive linkage in CRC families (Abulí et al., manuscript in preparation). SNP selection in the mucin gene family was performed using Pupasuite, a web tool for the selection of genetic variants with potential phenotypic effect (pupasuite.bioinfo.cipf.es) [26]. SNPs were always prioritized if they were coding, evolutionary conserved in mouse and SNP minor allele frequency (MAF) was above 5%. Other selected SNPs with a putative regulatory effect were in promoter, intronic or 3'-UTR regions. A complete list of SNP and genes analyzed in the present study is detailed in Table 1.

**Table 1:** SNPs genotyped in EPICOLON from the previously identified category and the mucin gene family to evaluate their implication in CRC genetic susceptibility.

Gene	SNP ID	Chr	Position*	Category	SNP type	Alleles
*MTHFR*	rs1801133	1	11856378	Previously identified	Missense (A222V)	C/T
*IRS1*	rs1801278	2	227660544	Previously identified	Missense (G971R)	A/G
*PPARG*	rs1801282	3	12393125	Previously identified	Missense (P12A)	C/G
*IL8*	rs4073	4	74606024	Previously identified	Promoter	A/T
*ADH1C*	rs698	4	100260789	Previously identified	Missense (I350V)	A/G
*APC*	rs459552	5	112176756	Previously identified	Missense (V1822D)	A/T
*IL6*	rs1800795	7	22766645	Previously identified	Promoter	C/G
*CCDN1*	rs9344	11	69462910	Previously identified	Synonymous (P241P)	A/G
*VDR*	rs2228570	12	48272895	Previously identified	Missense (M1T)	C/T
*ARL11*	rs3803185	13	50205025	Previously identified	Missense (C158R)	A/G

*OVGP1*	rs10067	1	111957311	Mucin family	Missense (H604Q)	C/G
*GALNT14*	rs2288101	2	31135184	Mucin family	Missense (Q469K)	A/C
*GALNT14*	rs11676188	2	31352788	Mucin family	Intronic	C/G
*GALNTL2*	rs2102302	3	16215650	Mucin family	Promoter	A/G
*MUC13*	rs12732	3	124624568	Mucin family	3'-UTR	C/T
*MUC13*	rs4679392	3	124646594	Mucin family	Missense (I99T)	A/G
*MUC7*	rs6826961	4	71346701	Mucin family	Missense (N80K)	C/G
*GALNT10*	rs6580076	5	153783753	Mucin family	Synonymous (A382A)	C/T
*GALNT10*	rs2277937	5	153799165	Mucin family	3'-UTR	C/T
*MUC21*	rs1634730	6	30954245	Mucin family	Missense (V98A)	C/T
*MUC12*	rs11766125	7	100648117	Mucin family	Missense (T4758R)	C/G
*MUC17*	rs4729656	7	100701610	Mucin family	3'-UTR	A/T
*MUC15*	rs15783	11	26586801	Mucin family	Missense (T229I)	A/G
*MUC15*	rs11029621	11	26596998	Mucin family	3' near gene	A/G
*MUC19*	rs2933353	12	40857943	Mucin family	Missense (G1803A)	A/C
*GALNT4*	rs2230283	12	89916811	Mucin family	Missense (V506I)	A/G
*GALNT1*	rs17647532	18	33234072	Mucin family	Promoter	C/T
*MUC16*	rs1862458	19	9069792	Mucin family	Missense (S5885F)	A/G

SNP ID, single nucleotide polymorphism identification.

Chr: Chromosome; UTR: untranslated region. *According to NCBI build 37.1 available at http://www.ncbi.nlm.nih.gov/sites/entrez?db=snp.

High-throughput genotyping in EPICOLON cohorts was performed according to manufacturer's instructions with the TaqMan allelic discrimination and SNPlex™ systems (Applied Biosystems, Foster City, USA), and single-base primer extension chemistry matrix-assisted laser desorption/ionization time-of-flight mass spectrometry (MALDI-TOF MS) genotyping platform (Sequenom Inc., San Diego, USA). Genotyping in the MCC-Spain cohort was performed using the VeraCode technology (Illumina, San Diego, USA). Genotyping was performed at the Santiago de Compostela and Barcelona nodes of the Spanish National Genotyping Centre http://www.cegen.org, and at the Genome Analysis Platform of the CIC-BioGUNE http://www.cicbiogune.es.

### Statistical methods

As quality control, genotyping success was set above 90% for SNPs. Allelic frequency description and Hardy-Weinberg equilibrium test were performed using SNPator, a web-based tool offered by the Bioinformatics division of the Spanish National Genotyping Centre (bioinformatica.cegen.upf.es) [27]. All SNPs analyzed had a genotype success rate > 90%. The genotype frequencies of all variants in the control population fitted the Hardy-Weinberg equilibrium (*P *> 0.01), supporting absence of genotyping artifacts. There was no sign of underlying population stratification in EPICOLON as tested by an independent study [7]. Genotype analysis was carried out using the SNPassoc R library [28]. Inter-group comparisons of genotype frequency differences were performed by regression analysis for codominant, dominant, recessive and log-additive models of inheritance. We estimated the crude odds ratio (OR) and their 95% confidence intervals (95% CIs). As expected, results did not change after sex and age adjustment. The best genetic or inheritance model was selected using the Akaike information criteria. To address the issue of multiple testing, we used Bonferroni correction (*P *= 0.0125 for four SNPs). Study power was estimated with CaTS software [29].

## Results

Twenty-eight SNPs, ten from the previously identified category and 18 from the mucin gene family, were successfully genotyped in EPICOLON stage 1. Results in EPICOLON stage 1 are shown in Additional File 1. Four SNPs were significant with a *P*-value < 0.05 in any of the tested inheritance models: rs698 in *ADH1C *(OR = 1.63, 95% CI = 1.06-2.50, *P*-value = 0.02, recessive, AA-AG vs GG), rs1800795 in *IL6 *(OR = 1.62, 95% CI = 1.10-2.37, *P*-value = 0.01, recessive, GG-GC vs CC), rs3803185 in *ARL11 *(OR = 1.58, 95% CI = 1.17-2.15, *P*-value = 0.007, codominant, GG vs AG vs GG), and rs2102302 in *GALNTL2 *(OR = 1.20, 95% CI = 1.00-1.44, *P*-value = 0.04, log-additive 0, 1, 2 alleles).

In the EPICOLON stage 1 cohort, a liberal *P*-value threshold (*P*-value < 0.05) was used to avoid false-negative results. We then validated statistically-significant stage 1 results by replicating them in another independent CRC cohort (EPICOLON stage 2). Further replication in stage 2 was performed only for significant SNPs in stage 1. Results for rs698, rs1800795, rs3803185, and rs2102302 in EPICOLON stage 2 are shown in Additional File 2. Only rs3803185 maintained statistical significance in stage 2 (OR = 1.34, 95% CI = 1.06-1.69, *P*-value = 0.01, recessive model, AA-AG vs GG). In order to improve statistical power, results for EPICOLON stages 1 and 2 were also analyzed jointly for these four SNPs. Results were only significant for rs3803185 (OR = 1.12, 95% CI = 1.00-1.25, *P*-value = 0.04, log-additive 0, 1, 2 alleles) and borderline significant for rs698 and rs2102302 (Table 2).

**Table 2:** SNPassoc results for previously identified and mucin SNPs in EPICOLON cohorts.

EPICOLON stages 1+2 (1,416 CRC cases and 1,424 controls)								
***ADH1C***								
**rs698**	Controls	%	Cases	%	OR	lower	upper	*P*-value

Codominant								
A/A	629	46.1	616	44.2	1.00			0.13565
A/G	610	44.7	620	44.4	1.04	0.89	1.22	
G/G	125	9.2	159	11.4	1.30	1.00	1.69	
Dominant								
A/A	629	46.1	616	44.2	1.00			0.29181
A/G-G/G	735	53.9	779	55.8	1.08	0.93	1.26	
Recessive								
A/A-A/G	1239	90.8	1236	88.6	1.00			0.05243
G/G	125	9.2	159	11.4	1.28	1.00	1.63	
Log-Additive								
0, 1, 2	1364	49.4	1395	50.6	1.10	0.98	1.24	0.09043
								

***IL6***								
**rs1800795**	Controls	%	Cases	%	OR	lower	upper	*P*-value

Codominant								
G/G	593	42.7	586	41.7	1.00			0.7950
G/C	623	44.9	635	45.2	1.03	0.88	1.21	
C/C	172	12.4	184	13.1	1.08	0.85	1.37	
Dominant								
G/G	593	42.7	586	41.7	1.00			0.5862
G/C-C/C	795	57.3	819	58.3	1.04	0.90	1.21	
Recessive								
G/G-G/C	1216	87.6	1221	86.9	1.00			0.5763
C/C	172	12.4	184	13.1	1.07	0.85	1.33	
log-Additive								
0, 1, 2	1388	49.7	1405	50.3	1.04	0.93	1.16	0.5034
								

***ARL11***								
**rs3803185**	Controls	%	Cases	%	OR	lower	upper	*P*-value

Codominant								
A/A	359	26.9	301	23.7	1.00			0.12263
A/G	686	51.4	666	52.4	1.15	0.95	1.39	
G/G	289	21.7	304	23.9	1.26	1.01	1.57	
Dominant								
A/A	359	26.9	301	23.7	1.00			0.06417
A/G-G/G	975	73.1	970	76.3	1.18	0.99	1.41	
Recessive								
A/A-A/G	1045	78.3	967	76.1	1.00			0.15480
G/G	289	21.7	304	23.9	1.14	0.95	1.37	
log-Additive								
0, 1, 2	1334	51.2	1271	48.8	1.12	1.00	1.25	**0.04338**
								

***GALNTL2***								
**rs2102302**	Controls	%	Cases	%	OR	lower	upper	*P*-value

Codominant								
A/A	579	42.1	538	39.0	1.00			0.16056
A/G	607	44.1	624	45.3	1.11	0.94	1.30	
G/G	189	13.7	217	15.7	1.23	0.98	1.55	
Dominant								
A/A	579	42.1	538	39.0	1.00			0.0971
A/G-G/G	796	57.9	841	61.0	1.14	0.98	1.32	
Recessive								
A/A-A/G	1186	86.3	1162	84.3	1.00			0.14317
G/G	189	13.7	217	15.7	1.17	0.95	1.45	
log-Additive								
0, 1, 2	1375	49.9	1379	50.1	1.11	1.00	1.24	0.0558

Significant *P*-values (< 0.05) are highlighted in bold.

OR, odds ratio

In order to further investigate rs3803185, we were able to genotype it additionally outside EPICOLON in a cohort from a Spanish multicase-control population study for common neoplasms (MCC-Spain). This variant was not significantly associated with CRC risk in this independent cohort. Pooled analysis for rs3803185 in the EPICOLON and MCC-Spain cohorts still showed borderline significance (OR = 1.08, 95% CI = 0.98-1.18, *P*-value = 0.09, log-additive 0, 1, 2 alleles).

Associations of rs698, rs1800795, rs3803185 and rs2102302 were also evaluated in 2 cohorts described in a previous GWAS [30], either by checking the original variant or a proxy SNP highly correlated with it (r^2 ^> 0.7) (Table 3). Interestingly, rs3803185 showed again significance in one of the GWAS (*P *= 0.03). However, it should be commented that weak associations observed in our study would have not been present if Bonferroni correction for multiple testing was applied and, therefore, they should be considered as not statistically significant.

**Table 3:** Association results for four selected SNPs evaluated in two external GWAS (CORGI and VQ58), either by checking the original variant or a proxy SNP highly correlated with it (r^2 ^> 0

	Present in I5?	Present in I3?	Proxy	r^2 ^and D'	*P*-value (CORGI)	*P*-value (VQ58)
**rs698**	no	no	rs1789924	1.000 1.000	0.1806	0.1658
**rs1800795**	no	no	rs1554606	0.868 0.932	0.1743	0.3346
**rs3803185**	no	no	rs4942859	0.716 1.000	0.5943	**0.03525**
**rs2102302**	no	no	rs2730351	0.853 1.000	0.1447	0.4666

Significant *P*-values (< 0.05) are highlighted in bold.

I5, Illumina HumanHap550; I3 Illumina HumanHap300.

## Discussion

Ten previously identified SNPs in *ADH1C*, *APC*, *CCDN1*, *IL6*, *IL8*, *IRS1*, *MTHFR*, *PPARG*, *VDR *and *ARL11*, and 18 selected variants in the mucin gene family were evaluated with a genetic association strategy. CRC cases and matched controls collected in two independent stages within the EPICOLON consortium were genotyped in order to evaluate its potential association with CRC risk. Mucins and GALNTs, members of the mucin gene family, have not been previously evaluated for CRC genetic susceptibility.

Significant results for previously identified SNPs in independent cohorts regarding CRC risk were previously reported for *APC *[11,12], *CCDN1 *[13,14], *IL6 *and *IL8 *[15,16], *IRS1 *[17], *MTHFR *[18-20], *PPARG *[21], and *VDR *[22,23]. Remarkably, variants in *MTHFR *and *CCDN1 *were additionally supported by meta-analyses of several individual studies [31]. However, none of them was confirmed in our cohort.

In our study, four SNPs were significant in EPICOLON stage 1 (rs698 in *ADH1C*, rs1800795 in *IL6*, rs3803185 in *ARL11*, and rs2102302 in *GALNTL2*), but only rs3803185 achieved statistical significance in EPICOLON stage 2. In the joint analysis for both stages, results were only significant for rs3803185 and borderline significant for rs698 and rs2102302. The rs3803185 variant was not significantly associated with CRC risk in an external cohort (MCC-Spain), but it still showed some borderline significance in the pooled analysis of both cohorts.

*ARL11*, also known as *ARLTS1 *(ADP-ribosylation factor-like tumor suppressor gene 1), is a tumor suppressor gene that belongs to the ARF family of the Ras superfamily of small GTPases that are known to be involved in multiple regulatory pathways altered in human carcinogenesis. It has been suggested that *ARL11 *SNPs, especially rs3803185, may act as low penetrance variants in several neoplasms including CRC [6,24,32]. The *ADH1C *gene encodes for class I alcohol dehydrogenase, gamma subunit, which is a member of the alcohol dehydrogenase family. Members of this enzyme family metabolize a wide variety of substrates, including ethanol. There is a noticeable association between alcohol consumption and CRC risk [33], and it is plausible that this association could also be modified by germline variants in enzymes that metabolize ethanol. *GALNTL2 *(UDP-N-acetyl-alpha-D-galactosamine:polypeptide N-acetylgalactosaminyltransferase-like 2), also known as *GALNT15*, is ubiquitously expressed in human tissues [34]. This gene has never been investigated in CRC susceptibility and it has only been reported to be studied as a genetic factor involved in longevity [35]. Chronic inflammation is in the etiology of CRC and release of large amount of cytokines and growth factors may influence the carcinogenesis process. The *IL6 *(interleukin 6) gene, among others, has been analyzed in previous independent genetic association studies and it is an *a priori *attractive candidate for genetic susceptibility to CRC [15,16].

Finally, as limitations of our study, it should be commented that our cohort sample size may probably be not large enough to reach stronger conclusions for the analyzed variants. However, our study (1,416 CRC cases and 1,424 controls for the EPICOLON cohorts) had an estimated 80% power to detect an OR as low as 1.3 with a MAF of 0.30, and 1.34 for a MAF down to 0.20, assuming a log-additive model and α = 0.05. Also, since we did not follow a GWAS strategy, our results apply only for the selected SNPs. Nevertheless, gene/SNP selection was biased to include those with previously published positive association results for CRC risk or those mucin SNPs with a putative functional effect.

In summary, none of the 28 SNPs analyzed in our study could be associated with CRC risk. However, variants in *ARL11*, *ADH1C*, *GALNTL2 *and *IL6 *may have an effect on CRC risk. Mucins and GALNTs, included in the mucin gene family, have been found deregulated in CRC and other neoplasms, and they are interesting candidates for CRC genetic susceptibility [27]. Since they have not been previously evaluated and despite our mostly negative results, we consider that genetic variation in the mucin gene family should be further explored in larger CRC cohorts in order to draw more solid conclusions. Also, additional case-control studies in larger CRC cohorts and meta-analyses could be useful to confirm or refute the role of *ARL11*, *ADH1C*, *GALNTL2 *and *IL6 *variants in CRC susceptibility.

## Conclusions

None of the 28 SNPs analyzed in our study could be associated with CRC risk. However, *ARL11*, *ADH1C*, *GALNTL2 *and *IL6 *genetic variants may have an effect on CRC risk. Further validation and meta-analyses should be undertaken in larger CRC cohorts.

## Abbreviations

CRC: colorectal cancer; SNP: single nucleotide polymorphism; GWAS: genome-wide association study; GALNT: N-acetylgalactosaminyltransferase; MCC: multicase-control; MAF: minor allele frequency; MALDI-TOF MS: matrix-assisted laser desorption/ionization time-of-flight mass spectrometry; 95% CI: 95% confidence interval; OR: odds ratio; ARLTS1: ADP-ribosylation factor-like tumor suppressor gene 1; GALNTL2: UDP-N-acetyl-alpha-D-galactosamine:polypeptide N-acetylgalactosaminyltransferase-like 2; Chr: Chromosome; UTR: untranslated region; I5: Illumina HumanHap550; I3 Illumina HumanHap300; AIC: Akaike information content.

## Competing interests

The authors declare that they have no competing interests.

## Authors' contributions

Samples were collected within the EPICOLON by XB, DG, JCu, JCl, JDM, JR, ML, FF-B, EP, SR, RJ, XLl, VM, AC and MA and other members of this consortium. Study was designed by SCB and CRP. Data acquisition was performed by all authors. Quality control was performed by AA, CFR, VG, XB, RJ, XLL, MA and SCB. Data was analyzed and interpreted by AA, CFR, CRP and SCB. Statistical analysis was performed by AA, CV, VM and SCB. Manuscript was prepared by AA and SCB. All authors read and approved the final manuscript.

## Pre-publication history

The pre-publication history for this paper can be accessed here:

http://www.biomedcentral.com/1471-2407/11/339/prepub

## Supplementary Material

Additional file 1 **Results for previously identified and mucin SNPs in EPICOLON stage 1**. SNPassoc results for previously identified and mucin SNPs in EPICOLON stage 1. *P*-values for some SNPs are highlighted in bold if significant (*P *< 0.05). Click here for file

Additional file 2 **Results for previously identified and mucin SNPs in EPICOLON stage 2**. SNPassoc results for previously identified and mucin SNPs in EPICOLON stage 2. *P*-values for some SNPs are highlighted in bold if significant (*P *< 0.05). Click here for file
