# Supplementary material for: Case-control study for colorectal cancer genetic susceptibility in EPICOLON: previously identified variants and mucins
Source: BMC Cancer. 2011 Aug 5;11:339. doi: 10.1186/1471-2407-11-339 (PMC3176240; doi:10.1186/1471-2407-11-339)
Supplement: Additional file 1 — Results for previously identified and mucin SNPs in EPICOLON stage 1. SNPassoc results for previously identified and mucin SNPs in EPICOLON stage 1. P-values for some SNPs are highlighted in bold if significant (P < 0.05). [file 1471-2407-11-339-S1.DOC]

**Additional File 1.** SNPassoc results for previously identified and mucin SNPs in EPICOLON stage 1. *P*-values for some SNPs are highlighted in bold if significant (*P*< 0.05).

| **rs1801133** | Controls | % | Cases | % | OR | lower | upper | *P*-value | AIC |
| --- | --- | --- | --- | --- | --- | --- | --- | --- | --- |
| Codominant |  |  |  |  |  |  |  |  |  |
| C/C | 189 | 38.1 | 169 | 32.8 | 1.00 |  |  | 0.18656 | 1404 |
| C/T | 238 | 48.0 | 274 | 53.2 | 1.29 | 0.98 | 1.69 |  |  |
| T/T | 69 | 13.9 | 72 | 14.0 | 1.17 | 0.79 | 1.72 |  |  |
| Dominant |  |  |  |  |  |  |  |  |  |
| C/C | 189 | 38.1 | 169 | 32.8 | 1.00 |  |  | 0.07870 | 1402 |
| C/T-T/T | 307 | 61.9 | 346 | 67.2 | 1.26 | 0.97 | 1.63 |  |  |
| Recessive |  |  |  |  |  |  |  |  |  |
| C/C-C/T | 427 | 86.1 | 443 | 86.0 | 1.00 |  |  | 0.97464 | 1405 |
| T/T | 69 | 13.9 | 72 | 14.0 | 1.01 | 0.70 | 1.44 |  |  |
| log-Additive |  |  |  |  |  |  |  |  |  |
| 0,1,2 | 496 | 49.1 | 515 | 50.9 | 1.13 | 0.94 | 1.36 | 0.20276 | 1404 |
|  |  |  |  |  |  |  |  |  |  |
| **rs1801278** | Controls | % | Cases | % | OR | lower | upper | *P*-value | AIC |
| Codominant |  |  |  |  |  |  |  |  |  |
| G/G | 432 | 85.5 | 422 | 82.3 | 1.00 |  |  | 0.3461 | 1415 |
| A/G | 69 | 13.7 | 85 | 16.6 | 1.26 | 0.89 | 1.78 |  |  |
| A/A | 4 | 0.8 | 6 | 1.2 | 1.54 | 0.43 | 5.48 |  |  |
| Dominant |  |  |  |  |  |  |  |  |  |
| G/G | 432 | 85.5 | 422 | 82.3 | 1.00 |  |  | 0.1538 | 1413 |
| A/G-A/A | 73 | 14.5 | 91 | 17.7 | 1.28 | 0.91 | 1.79 |  |  |
| Recessive |  |  |  |  |  |  |  |  |  |
| G/G-A/G | 501 | 99.2 | 507 | 98.8 | 1.00 |  |  | 0.5400 | 1415 |
| A/A | 4 | 0.8 | 6 | 1.2 | 1.48 | 0.42 | 5.28 |  |  |
| log-Additive |  |  |  |  |  |  |  |  |  |
| 0,1,2 | 505 | 49.6 | 513 | 50.4 | 1.26 | 0.92 | 1.71 | 0.1454 | 1413 |
|  |  |  |  |  |  |  |  |  |  |
| **rs1801282** | Controls | % | Cases | % | OR | lower | upper | *P*-value | AIC |
| Codominant |  |  |  |  |  |  |  |  |  |
| C/C | 419 | 83.5 | 426 | 82.7 | 1.00 |  |  | 0.8242 | 1415 |
| C/G | 80 | 15.9 | 87 | 16.9 | 1.07 | 0.77 | 1.49 |  |  |
| G/G | 3 | 0.6 | 2 | 0.4 | 0.66 | 0.11 | 3.94 |  |  |
| Dominant |  |  |  |  |  |  |  |  |  |
| C/C | 419 | 83.5 | 426 | 82.7 | 1.00 |  |  | 0.7504 | 1414 |
| C/G-G/G | 83 | 16.5 | 89 | 17.3 | 1.05 | 0.76 | 1.46 |  |  |
| Recessive |  |  |  |  |  |  |  |  |  |
| C/C-C/G | 499 | 99.4 | 513 | 99.6 | 1.00 |  |  | 0.6324 | 1414 |
| G/G | 3 | 0.6 | 2 | 0.4 | 0.65 | 0.11 | 3.90 |  |  |
| log-Additive |  |  |  |  |  |  |  |  |  |
| 0,1,2 | 502 | 49.4 | 515 | 50.6 | 1.04 | 0.76 | 1.42 | 0.8266 | 1414 |
|  |  |  |  |  |  |  |  |  |  |
| **rs4073** | Controls | % | Cases | % | OR | lower | upper | *P*-value | AIC |
| Codominant |  |  |  |  |  |  |  |  |  |
| T/T | 154 | 30.5 | 163 | 32.1 | 1.00 |  |  | 0.3048 | 1406 |
| A/T | 243 | 48.1 | 255 | 50.3 | 0.99 | 0.75 | 1.31 |  |  |
| A/A | 108 | 21.4 | 89 | 17.6 | 0.78 | 0.54 | 1.11 |  |  |
| Dominant |  |  |  |  |  |  |  |  |  |
| T/T | 154 | 30.5 | 163 | 32.1 | 1.00 |  |  | 0.5703 | 1407 |
| A/T-A/A | 351 | 69.5 | 344 | 67.9 | 0.93 | 0.71 | 1.21 |  |  |
| Recessive |  |  |  |  |  |  |  |  |  |
| T/T-A/T | 397 | 78.6 | 418 | 82.4 | 1.00 |  |  | 0.1235 | 1405 |
| A/A | 108 | 21.4 | 89 | 17.6 | 0.78 | 0.57 | 1.07 |  |  |
| log-Additive |  |  |  |  |  |  |  |  |  |
| 0,1,2 | 505 | 49.9 | 507 | 50.1 | 0.89 | 0.75 | 1.07 | 0.2142 | 1405 |
|  |  |  |  |  |  |  |  |  |  |
| **rs698** | Controls | % | Cases | % | OR | lower | upper | *P*-value | AIC |
| Codominant |  |  |  |  |  |  |  |  |  |
| A/A | 237 | 48.0 | 253 | 49.1 | 1.00 |  |  | **0.04160** | 1398 |
| A/G | 220 | 44.5 | 202 | 39.2 | 0.86 | 0.66 | 1.12 |  |  |
| G/G | 37 | 7.5 | 60 | 11.7 | 1.52 | 0.97 | 2.37 |  |  |
| Dominant |  |  |  |  |  |  |  |  |  |
| A/A | 237 | 48.0 | 253 | 49.1 | 1.00 |  |  | 0.71471 | 1402 |
| A/G-G/G | 257 | 52.0 | 262 | 50.9 | 0.95 | 0.75 | 1.22 |  |  |
| Recessive |  |  |  |  |  |  |  |  |  |
| A/A-A/G | 457 | 92.5 | 455 | 88.3 | 1.00 |  |  | **0.02430** | 1397 |
| G/G | 37 | 7.5 | 60 | 11.7 | 1.63 | 1.06 | 2.50 |  |  |
| log-Additive |  |  |  |  |  |  |  |  |  |
| 0,1,2 | 494 | 49.0 | 515 | 51.0 | 1.07 | 0.89 | 1.29 | 0.46600 | 1402 |
|  |  |  |  |  |  |  |  |  |  |
| **rs459552** | Controls | % | Cases | % | OR | lower | upper | *P*-value | AIC |
| Codominant |  |  |  |  |  |  |  |  |  |
| A/A | 322 | 63.3 | 342 | 66.4 | 1.00 |  |  | 0.1798 | 1422 |
| A/T | 164 | 32.2 | 160 | 31.1 | 0.92 | 0.70 | 1.20 |  |  |
| T/T | 23 | 4.5 | 13 | 2.5 | 0.53 | 0.27 | 1.07 |  |  |
| Dominant |  |  |  |  |  |  |  |  |  |
| A/A | 322 | 63.3 | 342 | 66.4 | 1.00 |  |  | 0.2917 | 1422 |
| A/T-T/T | 187 | 36.7 | 173 | 33.6 | 0.87 | 0.67 | 1.13 |  |  |
| Recessive |  |  |  |  |  |  |  |  |  |
| A/A-A/T | 486 | 95.5 | 502 | 97.5 | 1.00 |  |  | 0.0813 | 1420 |
| T/T | 23 | 4.5 | 13 | 2.5 | 0.55 | 0.27 | 1.09 |  |  |
| log-Additive |  |  |  |  |  |  |  |  |  |
| 0,1,2 | 509 | 49.7 | 515 | 50.3 | 0.85 | 0.68 | 1.06 | 0.1378 | 1421 |
|  |  |  |  |  |  |  |  |  |  |
| **rs1800795** | Controls | % | Cases | % | OR | lower | upper | *P*-value | AIC |
| Codominant |  |  |  |  |  |  |  |  |  |
| G/G | 231 | 45.6 | 211 | 41.0 | 1.00 |  |  | **0.03468** | 1416 |
| C/G | 227 | 44.8 | 228 | 44.3 | 1.10 | 0.85 | 1.43 |  |  |
| C/C | 49 | 9.7 | 76 | 14.8 | 1.70 | 1.13 | 2.55 |  |  |
| Dominant |  |  |  |  |  |  |  |  |  |
| G/G | 231 | 45.6 | 211 | 41.0 | 1.00 |  |  | 0.13847 | 1418 |
| C/G-C/C | 276 | 54.4 | 304 | 59.0 | 1.21 | 0.94 | 1.54 |  |  |
| Recessive |  |  |  |  |  |  |  |  |  |
| G/G-C/G | 458 | 90.3 | 439 | 85.2 | 1.00 |  |  | **0.01265** | 1414 |
| C/C | 49 | 9.7 | 76 | 14.8 | 1.62 | 1.10 | 2.37 |  |  |
| log-Additive |  |  |  |  |  |  |  |  |  |
| 0,1,2 | 507 | 49.6 | 515 | 50.4 | 1.24 | 1.03 | 1.48 | **0.02213** | 1416 |
|  |  |  |  |  |  |  |  |  |  |
| **rs9344** | Controls | % | Cases | % | OR | lower | upper | *P*-value | AIC |
| Codominant |  |  |  |  |  |  |  |  |  |
| A/A | 122 | 24.4 | 136 | 26.4 | 1.00 |  |  | 0.4534 | 1410 |
| A/G | 260 | 52.1 | 248 | 48.2 | 0.86 | 0.63 | 1.16 |  |  |
| G/G | 117 | 23.4 | 131 | 25.4 | 1.00 | 0.71 | 1.42 |  |  |
| Dominant |  |  |  |  |  |  |  |  |  |
| A/A | 122 | 24.4 | 136 | 26.4 | 1.00 |  |  | 0.4739 | 1409 |
| A/G-G/G | 377 | 75.6 | 379 | 73.6 | 0.90 | 0.68 | 1.20 |  |  |
| Recessive |  |  |  |  |  |  |  |  |  |
| A/A-A/G | 382 | 76.6 | 384 | 74.6 | 1.00 |  |  | 0.4610 | 1409 |
| G/G | 117 | 23.4 | 131 | 25.4 | 1.11 | 0.84 | 1.48 |  |  |
| log-Additive |  |  |  |  |  |  |  |  |  |
| 0,1,2 | 499 | 49.2 | 515 | 50.8 | 1.00 | 0.84 | 1.19 | 0.9944 | 1409 |
|  |  |  |  |  |  |  |  |  |  |
| **rs2228570** | Controls | % | Cases | % | OR | lower | upper | *P*-value | AIC |
| Codominant |  |  |  |  |  |  |  |  |  |
| C/C | 217 | 43.0 | 224 | 43.5 | 1.00 |  |  | 0.9304 | 1420 |
| C/T | 228 | 45.1 | 227 | 44.1 | 0.96 | 0.74 | 1.25 |  |  |
| T/T | 60 | 11.9 | 64 | 12.4 | 1.03 | 0.69 | 1.54 |  |  |
| Dominant |  |  |  |  |  |  |  |  |  |
| C/C | 217 | 43.0 | 224 | 43.5 | 1.00 |  |  | 0.8657 | 1418 |
| C/T-T/T | 288 | 57.0 | 291 | 56.5 | 0.98 | 0.76 | 1.25 |  |  |
| Recessive |  |  |  |  |  |  |  |  |  |
| C/C-C/T | 445 | 88.1 | 451 | 87.6 | 1.00 |  |  | 0.7896 | 1418 |
| T/T | 60 | 11.9 | 64 | 12.4 | 1.05 | 0.72 | 1.53 |  |  |
| log-Additive |  |  |  |  |  |  |  |  |  |
| 0,1,2 | 505 | 49.5 | 515 | 50.5 | 1.00 | 0.83 | 1.20 | 0.9960 | 1418 |
|  |  |  |  |  |  |  |  |  |  |
| **rs3803185** | Controls | % | Cases | % | OR | lower | upper | *P*-value | AIC |
| Codominant |  |  |  |  |  |  |  |  |  |
| T/T | 135 | 26.2 | 100 | 19.4 | 1.00 |  |  | **0.007859** | 1424 |
| C/T | 270 | 52.4 | 317 | 61.6 | 1.58 | 1.17 | 2.15 |  |  |
| C/C | 110 | 21.4 | 98 | 19.0 | 1.20 | 0.83 | 1.75 |  |  |
| Dominant |  |  |  |  |  |  |  |  |  |
| T/T | 135 | 26.2 | 100 | 19.4 | 1.00 |  |  | **0.00925** | 2 1425 |
| C/T-C/C | 380 | 73.8 | 415 | 80.6 | 1.47 | 1.10 | 1.98 |  |  |
| Recessive |  |  |  |  |  |  |  |  |  |
| T/T-C/T | 405 | 78.6 | 417 | 81.0 | 1.00 |  |  | 0.35154 | 3 1431 |
| C/C | 110 | 21.4 | 98 | 19.0 | 0.87 | 0.64 | 1.17 |  |  |
| log-Additive |  |  |  |  |  |  |  |  |  |
| 0,1,2 | 515 | 50.0 | 515 | 50.0 | 1.11 | 0.92 | 1.34 | 0.27400 | 6 1431 |
|  |  |  |  |  |  |  |  |  |  |
| **rs10067** | Controls | % | Cases | % | OR | lower | upper | *P*-value | AIC |
| Codominant |  |  |  |  |  |  |  |  |  |
| G/G | 380 | 75.0 | 380 | 75.2 | 1.00 |  |  | 0.2648 | 1406 |
| C/G | 110 | 21.7 | 116 | 23.0 | 1.05 | 0.78 | 1.42 |  |  |
| C/C | 17 | 3.4 | 9 | 1.8 | 0.53 | 0.23 | 1.20 |  |  |
| Dominant |  |  |  |  |  |  |  |  |  |
| G/G | 380 | 75.0 | 380 | 75.2 | 1.00 |  |  | 0.9131 | 1407 |
| C/G-C/C | 127 | 25.0 | 125 | 24.8 | 0.98 | 0.74 | 1.31 |  |  |
| Recessive |  |  |  |  |  |  |  |  |  |
| G/G-C/G | 490 | 96.6 | 496 | 98.2 | 1.00 |  |  | 0.1114 | 1404 |
| C/C | 17 | 3.4 | 9 | 1.8 | 0.52 | 0.23 | 1.18 |  |  |
| log-Additive |  |  |  |  |  |  |  |  |  |
| 0,1,2 | 507 | 50.1 | 505 | 49.9 | 0.93 | 0.73 | 1.19 | 0.5528 | 1407 |
|  |  |  |  |  |  |  |  |  |  |
| **rs2288101** | Controls | % | Cases | % | OR | lower | upper | *P*-value | AIC |
| Codominant |  |  |  |  |  |  |  |  |  |
| C/C | 296 | 58.7 | 283 | 57.5 | 1.00 |  |  | 0.8559 | 1386 |
| A/C | 180 | 35.7 | 178 | 36.2 | 1.03 | 0.79 | 1.35 |  |  |
| A/A | 28 | 5.6 | 31 | 6.3 | 1.16 | 0.68 | 1.98 |  |  |
| Dominant |  |  |  |  |  |  |  |  |  |
| C/C | 296 | 58.7 | 283 | 57.5 | 1.00 |  |  | 0.6988 | 1384 |
| A/C-A/A | 208 | 41.3 | 209 | 42.5 | 1.05 | 0.82 | 1.35 |  |  |
| Recessive |  |  |  |  |  |  |  |  |  |
| C/C-A/C | 476 | 94.4 | 461 | 93.7 | 1.00 |  |  | 0.6184 | 1384 |
| A/A | 28 | 5.6 | 31 | 6.3 | 1.14 | 0.68 | 1.94 |  |  |
| log-Additive |  |  |  |  |  |  |  |  |  |
| 0,1,2 | 504 | 50.6 | 492 | 49.4 | 1.05 | 0.86 | 1.29 | 0.6111 | 1384 |
|  |  |  |  |  |  |  |  |  |  |
| **rs11676188** | Controls | % | Cases | % | OR | lower | upper | *P*-value | AIC |
| Codominant |  |  |  |  |  |  |  |  |  |
| C/C | 319 | 62.9 | 336 | 68.0 | 1.00 |  |  | 0.23483 | 1391 |
| C/G | 164 | 32.3 | 137 | 27.7 | 0.79 | 0.60 | 1.04 |  |  |
| G/G | 24 | 4.7 | 21 | 4.3 | 0.83 | 0.45 | 1.52 |  |  |
| Dominant |  |  |  |  |  |  |  |  |  |
| C/C | 319 | 62.9 | 336 | 68.0 | 1.00 |  |  | 0.08986 | 1389 |
| C/G-G/G | 188 | 37.1 | 158 | 32.0 | 0.80 | 0.61 | 1.04 |  |  |
| Recessive |  |  |  |  |  |  |  |  |  |
| C/C-C/G | 483 | 95.3 | 473 | 95.7 | 1.00 |  |  | 0.71237 | 1391 |
| G/G | 24 | 4.7 | 21 | 4.3 | 0.89 | 0.49 | 1.63 |  |  |
| log-Additive |  |  |  |  |  |  |  |  |  |
| 0,1,2 | 507 | 50.6 | 494 | 49.4 | 0.84 | 0.68 | 1.05 | 0.12292 | 1389 |
|  |  |  |  |  |  |  |  |  |  |
| **rs2102302** | Controls | % | Cases | % | OR | lower | upper | *P*-value | AIC |
| Codominant |  |  |  |  |  |  |  |  |  |
| A/A | 214 | 42.4 | 185 | 37.9 | 1.00 |  |  | 0.12132 | 1378 |
| A/G | 230 | 45.5 | 224 | 45.9 | 1.13 | 0.86 | 1.47 |  |  |
| G/G | 61 | 12.1 | 79 | 16.2 | 1.50 | 1.02 | 2.21 |  |  |
| Dominant |  |  |  |  |  |  |  |  |  |
| A/A | 214 | 42.4 | 185 | 37.9 | 1.00 |  |  | 0.15111 | 1378 |
| A/G-G/G | 291 | 57.6 | 303 | 62.1 | 1.20 | 0.93 | 1.55 |  |  |
| Recessive |  |  |  |  |  |  |  |  |  |
| A/A-A/G | 444 | 87.9 | 409 | 83.8 | 1.00 |  |  | 0.06264 | 1377 |
| G/G | 61 | 12.1 | 79 | 16.2 | 1.41 | 0.98 | 2.02 |  |  |
| log-Additive |  |  |  |  |  |  |  |  |  |
| 0,1,2 | 505 | 50.9 | 488 | 49.1 | 1.20 | 1.00 | 1.44 | **0.04979** | 1376 |
|  |  |  |  |  |  |  |  |  |  |
| **rs12732** | Controls | % | Cases | % | OR | lower | upper | *P*-value | AIC |
| Codominant |  |  |  |  |  |  |  |  |  |
| T/T | 314 | 61.8 | 311 | 61.7 | 1.00 |  |  | 0.6836 | 1408 |
| C/T | 174 | 34.3 | 178 | 35.3 | 1.03 | 0.80 | 1.34 |  |  |
| C/C | 20 | 3.9 | 15 | 3.0 | 0.76 | 0.38 | 1.51 |  |  |
| Dominant |  |  |  |  |  |  |  |  |  |
| T/T | 314 | 61.8 | 311 | 61.7 | 1.00 |  |  | 0.9727 | 1407 |
| C/T-C/C | 194 | 38.2 | 193 | 38.3 | 1.00 | 0.78 | 1.29 |  |  |
| Recessive |  |  |  |  |  |  |  |  |  |
| T/T-C/T | 488 | 96.1 | 489 | 97.0 | 1.00 |  |  | 0.4021 | 1406 |
| C/C | 20 | 3.9 | 15 | 3.0 | 0.75 | 0.38 | 1.48 |  |  |
| log-Additive |  |  |  |  |  |  |  |  |  |
| 0,1,2 | 508 | 50.2 | 504 | 49.8 | 0.97 | 0.78 | 1.21 | 0.8075 | 1407 |
|  |  |  |  |  |  |  |  |  |  |
| **rs4679392** | Controls | % | Cases | % | OR | lower | upper | *P*-value | AIC |
| Codominant |  |  |  |  |  |  |  |  |  |
| A/A | 163 | 32.1 | 170 | 34.5 | 1.00 |  |  | 0.3635 | 1390 |
| A/G | 256 | 50.5 | 253 | 51.3 | 0.95 | 0.72 | 1.25 |  |  |
| G/G | 88 | 17.4 | 70 | 14.2 | 0.76 | 0.52 | 1.12 |  |  |
| Dominant |  |  |  |  |  |  |  |  |  |
| A/A | 163 | 32.1 | 170 | 34.5 | 1.00 |  |  | 0.4339 | 1390 |
| A/G-G/G | 344 | 67.9 | 323 | 65.5 | 0.90 | 0.69 | 1.17 |  |  |
| Recessive |  |  |  |  |  |  |  |  |  |
| A/A-A/G | 419 | 82.6 | 423 | 85.8 | 1.00 |  |  | 0.1706 | 1388 |
| G/G | 88 | 17.4 | 70 | 14.2 | 0.79 | 0.56 | 1.11 |  |  |
| log-Additive |  |  |  |  |  |  |  |  |  |
| 0,1,2 | 507 | 50.7 | 493 | 49.3 | 0.89 | 0.74 | 1.07 | 0.2006 | 1388 |
|  |  |  |  |  |  |  |  |  |  |
| **rs6826961** | Controls | % | Cases | % | OR | lower | upper | *P*-value | AIC |
| Codominant |  |  |  |  |  |  |  |  |  |
| C/C | 319 | 63.0 | 302 | 61.5 | 1.00 |  |  | 0.8665 | 1388 |
| C/G | 160 | 31.6 | 163 | 33.2 | 1.08 | 0.82 | 1.41 |  |  |
| G/G | 27 | 5.3 | 26 | 5.3 | 1.02 | 0.58 | 1.78 |  |  |
| Dominant |  |  |  |  |  |  |  |  |  |
| C/C | 319 | 63.0 | 302 | 61.5 | 1.00 |  |  | 0.6168 | 1386 |
| C/G-G/G | 187 | 37.0 | 189 | 38.5 | 1.07 | 0.83 | 1.38 |  |  |
| Recessive |  |  |  |  |  |  |  |  |  |
| C/C-C/G | 479 | 94.7 | 465 | 94.7 | 1.00 |  |  | 0.9772 | 1386 |
| G/G | 27 | 5.3 | 26 | 5.3 | 0.99 | 0.57 | 1.73 |  |  |
| log-Additive |  |  |  |  |  |  |  |  |  |
| 0,1,2 | 506 | 50.8 | 491 | 49.2 | 1.04 | 0.85 | 1.29 | 0.6904 | 1386 |
|  |  |  |  |  |  |  |  |  |  |
| **rs6580076** | Controls | % | Cases | % | OR | lower | upper | *P*-value | AIC |
| Codominant |  |  |  |  |  |  |  |  |  |
| C/C | 319 | 65.5 | 333 | 70.4 | 1.00 |  |  | 0.2585 | 1334 |
| C/T | 151 | 31.0 | 127 | 26.8 | 0.81 | 0.61 | 1.07 |  |  |
| T/T | 17 | 3.5 | 13 | 2.7 | 0.73 | 0.35 | 1.53 |  |  |
| Dominant |  |  |  |  |  |  |  |  |  |
| C/C | 319 | 65.5 | 333 | 70.4 | 1.00 |  |  | 0.1038 | 1332 |
| C/T-T/T | 168 | 34.5 | 140 | 29.6 | 0.80 | 0.61 | 1.05 |  |  |
| Recessive |  |  |  |  |  |  |  |  |  |
| C/C-C/T | 470 | 96.5 | 460 | 97.3 | 1.00 |  |  | 0.5080 | 1334 |
| T/T | 17 | 3.5 | 13 | 2.7 | 0.78 | 0.38 | 1.63 |  |  |
| log-Additive |  |  |  |  |  |  |  |  |  |
| 0,1,2 | 487 | 50.7 | 473 | 49.3 | 0.82 | 0.65 | 1.04 | 0.1047 | 1332 |
|  |  |  |  |  |  |  |  |  |  |
| **rs2277937** | Controls | % | Cases | % | OR | lower | upper | *P*-value | AIC |
| Codominant |  |  |  |  |  |  |  |  |  |
| T/T | 271 | 53.6 | 251 | 50.6 | 1.00 |  |  | 0.5831 | 1394 |
| C/T | 191 | 37.7 | 203 | 40.9 | 1.15 | 0.88 | 1.49 |  |  |
| C/C | 44 | 8.7 | 42 | 8.5 | 1.03 | 0.65 | 1.63 |  |  |
| Dominant |  |  |  |  |  |  |  |  |  |
| T/T | 271 | 53.6 | 251 | 50.6 | 1.00 |  |  | 0.3496 | 1392 |
| C/T-C/C | 235 | 46.4 | 245 | 49.4 | 1.13 | 0.88 | 1.44 |  |  |
| Recessive |  |  |  |  |  |  |  |  |  |
| T/T-C/T | 462 | 91.3 | 454 | 91.5 | 1.00 |  |  | 0.8975 | 1393 |
| C/C | 44 | 8.7 | 42 | 8.5 | 0.97 | 0.62 | 1.51 |  |  |
| log-Additive |  |  |  |  |  |  |  |  |  |
| 0,1,2 | 506 | 50.5 | 496 | 49.5 | 1.07 | 0.88 | 1.29 | 0.5045 | 1392 |
|  |  |  |  |  |  |  |  |  |  |
| **rs1634730** | Controls | % | Cases | % | OR | lower | upper | *P*-value | AIC |
| Codominant |  |  |  |  |  |  |  |  |  |
| C/C | 304 | 59.8 | 318 | 63.1 | 1.00 |  |  | 0.3641 | 1407 |
| C/T | 172 | 33.9 | 163 | 32.3 | 0.91 | 0.69 | 1.18 |  |  |
| T/T | 32 | 6.3 | 23 | 4.6 | 0.69 | 0.39 | 1.20 |  |  |
| Dominant |  |  |  |  |  |  |  |  |  |
| C/C | 304 | 59.8 | 318 | 63.1 | 1.00 |  |  | 0.2877 | 1406 |
| C/T-T/T | 204 | 40.2 | 186 | 36.9 | 0.87 | 0.68 | 1.12 |  |  |
| Recessive |  |  |  |  |  |  |  |  |  |
| C/C-C/T | 476 | 93.7 | 481 | 95.4 | 1.00 |  |  | 0.2223 | 1405 |
| T/T | 32 | 6.3 | 23 | 4.6 | 0.71 | 0.41 | 1.23 |  |  |
| log-Additive |  |  |  |  |  |  |  |  |  |
| 0,1,2 | 508 | 50.2 | 504 | 49.8 | 0.87 | 0.71 | 1.07 | 0.1828 | 1405 |
|  |  |  |  |  |  |  |  |  |  |
| **rs11766125** | Controls | % | Cases | % | OR | lower | upper | *P*-value | AIC |
| Codominant |  |  |  |  |  |  |  |  |  |
| C/C | 144 | 28.9 | 130 | 26.4 | 1.00 |  |  | 0.3520 | 1376 |
| C/G | 246 | 49.4 | 237 | 48.2 | 1.07 | 0.79 | 1.44 |  |  |
| G/G | 108 | 21.7 | 125 | 25.4 | 1.28 | 0.90 | 1.82 |  |  |
| Dominant |  |  |  |  |  |  |  |  |  |
| C/C | 144 | 28.9 | 130 | 26.4 | 1.00 |  |  | 0.3806 | 1376 |
| C/G-G/G | 354 | 71.1 | 362 | 73.6 | 1.13 | 0.86 | 1.50 |  |  |
| Recessive |  |  |  |  |  |  |  |  |  |
| C/C-C/G | 390 | 78.3 | 367 | 74.6 | 1.00 |  |  | 0.1676 | 1374 |
| G/G | 108 | 21.7 | 125 | 25.4 | 1.23 | 0.92 | 1.65 |  |  |
| log-Additive |  |  |  |  |  |  |  |  |  |
| 0,1,2 | 498 | 50.3 | 492 | 49.7 | 1.13 | 0.95 | 1.35 | 0.1712 | 1374 |
|  |  |  |  |  |  |  |  |  |  |
| **rs4729656** | Controls | % | Cases | % | OR | lower | upper | *P*-value | AIC |
| Codominant |  |  |  |  |  |  |  |  |  |
| A/A | 175 | 34.5 | 183 | 36.7 | 1.00 |  |  | 0.5573 | 1398 |
| A/T | 244 | 48.1 | 240 | 48.2 | 0.94 | 0.72 | 1.24 |  |  |
| T/T | 88 | 17.4 | 75 | 15.1 | 0.82 | 0.56 | 1.18 |  |  |
| Dominant |  |  |  |  |  |  |  |  |  |
| A/A | 175 | 34.5 | 183 | 36.7 | 1.00 |  |  | 0.4604 | 1397 |
| A/T-T/T | 332 | 65.5 | 315 | 63.3 | 0.91 | 0.70 | 1.17 |  |  |
| Recessive |  |  |  |  |  |  |  |  |  |
| A/A-A/T | 419 | 82.6 | 423 | 84.9 | 1.00 |  |  | 0.3231 | 1396 |
| T/T | 88 | 17.4 | 75 | 15.1 | 0.84 | 0.60 | 1.18 |  |  |
| log-Additive |  |  |  |  |  |  |  |  |  |
| 0,1,2 | 507 | 50.4 | 498 | 49.6 | 0.91 | 0.76 | 1.09 | 0.3006 | 1396 |
|  |  |  |  |  |  |  |  |  |  |
| **rs15783** | Controls | % | Cases | % | OR | lower | upper | *P*-value | AIC |
| Codominant |  |  |  |  |  |  |  |  |  |
| A/A | 248 | 49.0 | 221 | 44.4 | 1.00 |  |  | 0.2889 | 1395 |
| A/G | 209 | 41.3 | 219 | 44.0 | 1.18 | 0.90 | 1.53 |  |  |
| G/G | 49 | 9.7 | 58 | 11.6 | 1.33 | 0.87 | 2.02 |  |  |
| Dominant |  |  |  |  |  |  |  |  |  |
| A/A | 248 | 49.0 | 221 | 44.4 | 1.00 |  |  | 0.1411 | 1394 |
| A/G-G/G | 258 | 51.0 | 277 | 55.6 | 1.20 | 0.94 | 1.54 |  |  |
| Recessive |  |  |  |  |  |  |  |  |  |
| A/A-A/G | 457 | 90.3 | 440 | 88.4 | 1.00 |  |  | 0.3134 | 1395 |
| G/G | 49 | 9.7 | 58 | 11.6 | 1.23 | 0.82 | 1.84 |  |  |
| log-Additive |  |  |  |  |  |  |  |  |  |
| 0,1,2 | 506 | 50.4 | 498 | 49.6 | 1.16 | 0.96 | 1.40 | 0.1165 | 1393 |
|  |  |  |  |  |  |  |  |  |  |
| **rs11029621** | Controls | % | Cases | % | OR | lower | upper | *P*-value | AIC |
| Codominant |  |  |  |  |  |  |  |  |  |
| G/G | 129 | 25.8 | 134 | 27.5 | 1.00 |  |  | 0.6617 | 1373 |
| A/G | 236 | 47.2 | 233 | 47.8 | 0.95 | 0.70 | 1.29 |  |  |
| A/A | 135 | 27.0 | 120 | 24.6 | 0.86 | 0.61 | 1.21 |  |  |
| Dominant |  |  |  |  |  |  |  |  |  |
| G/G | 129 | 25.8 | 134 | 27.5 | 1.00 |  |  | 0.5422 | 1372 |
| A/G-A/A | 371 | 74.2 | 353 | 72.5 | 0.92 | 0.69 | 1.21 |  |  |
| Recessive |  |  |  |  |  |  |  |  |  |
| G/G-A/G | 365 | 73.0 | 367 | 75.4 | 1.00 |  |  | 0.3971 | 1371 |
| A/A | 135 | 27.0 | 120 | 24.6 | 0.88 | 0.66 | 1.18 |  |  |
| log-Additive |  |  |  |  |  |  |  |  |  |
| 0,1,2 | 500 | 50.7 | 487 | 49.3 | 0.93 | 0.78 | 1.10 | 0.3769 | 1371 |
|  |  |  |  |  |  |  |  |  |  |
| **rs2933353** | Controls | % | Cases | % | OR | lower | upper | *P*-value | AIC |
| Codominant |  |  |  |  |  |  |  |  |  |
| C/C | 286 | 56.4 | 284 | 57.0 | 1.00 |  |  | 0.9449 | 1399 |
| A/C | 201 | 39.6 | 193 | 38.8 | 0.97 | 0.75 | 1.25 |  |  |
| A/A | 20 | 3.9 | 21 | 4.2 | 1.06 | 0.56 | 1.99 |  |  |
| Dominant |  |  |  |  |  |  |  |  |  |
| C/C | 286 | 56.4 | 284 | 57.0 | 1.00 |  |  | 0.8433 | 1397 |
| A/C-A/A | 221 | 43.6 | 214 | 43.0 | 0.98 | 0.76 | 1.25 |  |  |
| Recessive |  |  |  |  |  |  |  |  |  |
| C/C-A/C | 487 | 96.1 | 477 | 95.8 | 1.00 |  |  | 0.8274 | 1397 |
| A/A | 20 | 3.9 | 21 | 4.2 | 1.07 | 0.57 | 2.00 |  |  |
| log-Additive |  |  |  |  |  |  |  |  |  |
| 0,1,2 | 507 | 50.4 | 498 | 49.6 | 0.99 | 0.80 | 1.23 | 0.9241 | 1397 |
|  |  |  |  |  |  |  |  |  |  |
| **rs2230283** | Controls | % | Cases | % | OR | lower | upper | *P*-value | AIC |
| Codominant |  |  |  |  |  |  |  |  |  |
| G/G | 210 | 41.7 | 213 | 45.7 | 1.00 |  |  | 0.4576 | 1346 |
| A/G | 232 | 46.1 | 199 | 42.7 | 0.85 | 0.65 | 1.11 |  |  |
| A/A | 61 | 12.1 | 54 | 11.6 | 0.87 | 0.58 | 1.32 |  |  |
| Dominant |  |  |  |  |  |  |  |  |  |
| G/G | 210 | 41.7 | 213 | 45.7 | 1.00 |  |  | 0.2144 | 1344 |
| A/G-A/A | 293 | 58.3 | 253 | 54.3 | 0.85 | 0.66 | 1.10 |  |  |
| Recessive |  |  |  |  |  |  |  |  |  |
| G/G-A/G | 442 | 87.9 | 412 | 88.4 | 1.00 |  |  | 0.7953 | 1346 |
| A/A | 61 | 12.1 | 54 | 11.6 | 0.95 | 0.64 | 1.40 |  |  |
| log-Additive |  |  |  |  |  |  |  |  |  |
| 0,1,2 | 503 | 51.9 | 466 | 48.1 | 0.91 | 0.75 | 1.09 | 0.2991 | 1345 |
|  |  |  |  |  |  |  |  |  |  |
| **rs17647532** | Controls | % | Cases | % | OR | lower | upper | *P*-value | AIC |
| Codominant |  |  |  |  |  |  |  |  |  |
| T/T | 370 | 73.0 | 370 | 74.3 | 1.00 |  |  | 0.6251 | 1398 |
| C/T | 128 | 25.2 | 116 | 23.3 | 0.91 | 0.68 | 1.21 |  |  |
| C/C | 9 | 1.8 | 12 | 2.4 | 1.33 | 0.56 | 3.20 |  |  |
| Dominant |  |  |  |  |  |  |  |  |  |
| T/T | 370 | 73.0 | 370 | 74.3 | 1.00 |  |  | 0.6352 | 1397 |
| C/T-C/C | 137 | 27.0 | 128 | 25.7 | 0.93 | 0.71 | 1.24 |  |  |
| Recessive |  |  |  |  |  |  |  |  |  |
| T/T-C/T | 498 | 98.2 | 486 | 97.6 | 1.00 |  |  | 0.4814 | 1397 |
| C/C | 9 | 1.8 | 12 | 2.4 | 1.37 | 0.57 | 3.27 |  |  |
| log-Additive |  |  |  |  |  |  |  |  |  |
| 0,1,2 | 507 | 50.4 | 498 | 49.6 | 0.97 | 0.76 | 1.25 | 0.8267 | 1397 |
|  |  |  |  |  |  |  |  |  |  |
| **rs1862458** | Controls | % | Cases | % | OR | lower | upper | *P*-value | AIC |
| Codominant |  |  |  |  |  |  |  |  |  |
| G/G | 354 | 70.0 | 357 | 71.8 | 1.00 |  |  | 0.7120 | 1396 |
| A/G | 134 | 26.5 | 126 | 25.4 | 0.93 | 0.70 | 1.24 |  |  |
| A/A | 18 | 3.6 | 14 | 2.8 | 0.77 | 0.38 | 1.57 |  |  |
| Dominant |  |  |  |  |  |  |  |  |  |
| G/G | 354 | 70.0 | 357 | 71.8 | 1.00 |  |  | 0.5144 | 1394 |
| A/G-A/A | 152 | 30.0 | 140 | 28.2 | 0.91 | 0.70 | 1.20 |  |  |
| Recessive |  |  |  |  |  |  |  |  |  |
| G/G-A/G | 488 | 96.4 | 483 | 97.2 | 1.00 |  |  | 0.5041 | 1394 |
| A/A | 18 | 3.6 | 14 | 2.8 | 0.79 | 0.39 | 1.60 |  |  |
| log-Additive |  |  |  |  |  |  |  |  |  |
| 0,1,2 | 506 | 50.4 | 497 | 49.6 | 0.91 | 0.72 | 1.15 | 0.4365 | 1394 |

OR, odds ratio; AIC, Akaike information content.
