# Supplementary material for: Case-control study for colorectal cancer genetic susceptibility in EPICOLON: previously identified variants and mucins
Source: BMC Cancer. 2011 Aug 5;11:339. doi: 10.1186/1471-2407-11-339 (PMC3176240; doi:10.1186/1471-2407-11-339)
Supplement: Additional file 2 — Results for previously identified and mucin SNPs in EPICOLON stage 2. SNPassoc results for previously identified and mucin SNPs in EPICOLON stage 2. P-values for some SNPs are highlighted in bold if significant (P < 0.05). [file 1471-2407-11-339-S2.DOC]

**Additional File 2.** SNPassoc results for previously identified and mucin SNPs in EPICOLON stage 2. *P*-values for some SNPs are highlighted in bold if significant (*P*< 0.05).

| **rs698** | Controls | % | Cases | % | OR | lower | upper | *P*-value | AIC |
| --- | --- | --- | --- | --- | --- | --- | --- | --- | --- |
| Codominant |  |  |  |  |  |  |  |  |  |
| A/A | 392 | 45.1 | 363 | 41.2 | 1.00 |  |  | 0.2625 | 2429 |
| A/G | 390 | 44.8 | 418 | 47.5 | 1.16 | 0.95 | 1.41 |  |  |
| G/G | 88 | 10.1 | 99 | 11.2 | 1.21 | 0.88 | 1.67 |  |  |
| Dominant |  |  |  |  |  |  |  |  |  |
| A/A | 392 | 45.1 | 363 | 41.2 | 1.00 |  |  | 0.1078 | 2427 |
| A/G-G/G | 478 | 54.9 | 517 | 58.8 | 1.17 | 0.97 | 1.41 |  |  |
| Recessive |  |  |  |  |  |  |  |  |  |
| A/A-A/G | 782 | 89.9 | 781 | 88.8 | 1.00 |  |  | 0.4421 | 2429 |
| G/G | 88 | 10.1 | 99 | 11.2 | 1.13 | 0.83 | 1.53 |  |  |
| Log-Additive |  |  |  |  |  |  |  |  |  |
| 0,1,2 | 870 | 49.7 | 880 | 50.3 | 1.12 | 0.97 | 1.29 | 0.1160 | 2428 |
|  |  |  |  |  |  |  |  |  |  |
| **rs1800795** | Controls | % | Cases | % | OR | lower | upper | *P*-value | AIC |
| Codominant |  |  |  |  |  |  |  |  |  |
| G/G | 362 | 41.1 | 375 | 42.1 | 1.00 |  |  | 0.5197 | 2460 |
| C/G | 396 | 44.9 | 407 | 45.7 | 0.99 | 0.81 | 1.21 |  |  |
| C/C | 123 | 14.0 | 108 | 12.1 | 0.85 | 0.63 | 1.14 |  |  |
| Dominant |  |  |  |  |  |  |  |  |  |
| G/G | 362 | 41.1 | 375 | 42.1 | 1.00 |  |  | 0.6555 | 2459 |
| C/G-C/C | 519 | 58.9 | 515 | 57.9 | 0.96 | 0.79 | 1.16 |  |  |
| Recessive |  |  |  |  |  |  |  |  |  |
| G/G-C/G | 758 | 86.0 | 782 | 87.9 | 1.00 |  |  | 0.2537 | 2458 |
| C/C | 123 | 14.0 | 108 | 12.1 | 0.85 | 0.65 | 1.12 |  |  |
| log-Additive |  |  |  |  |  |  |  |  |  |
| 0,1,2 | 881 | 49.7 | 890 | 50.3 | 0.94 | 0.82 | 1.08 | 0.3755 | 2458 |
|  |  |  |  |  |  |  |  |  |  |
| **rs3803185** | Controls | % | Cases | % | OR | lower | upper | *P*-value | AIC |
| Codominant |  |  |  |  |  |  |  |  |  |
| A/A | 224 | 27.4 | 201 | 26.6 | 1.00 |  |  | **0.03887** | 2180 |
| A/G | 416 | 50.8 | 349 | 46.2 | 0.93 | 0.74 | 1.19 |  |  |
| G/G | 179 | 21.9 | 206 | 27.2 | 1.28 | 0.97 | 1.69 |  |  |
| Dominant |  |  |  |  |  |  |  |  |  |
| A/A | 224 | 27.4 | 201 | 26.6 | 1.00 |  |  | 0.73316 | 2185 |
| A/G-G/G | 595 | 72.6 | 555 | 73.4 | 1.04 | 0.83 | 1.30 |  |  |
| Recessive |  |  |  |  |  |  |  |  |  |
| A/A-A/G | 640 | 78.1 | 550 | 72.8 | 1.00 |  |  | **0.01286** | 2179 |
| G/G | 179 | 21.9 | 206 | 27.2 | 1.34 | 1.06 | 1.69 |  |  |
| log-Additive |  |  |  |  |  |  |  |  |  |
| 0,1,2 | 819 | 52.0 | 756 | 48.0 | 1.13 | 0.98 | 1.29 | 0.08847 | 2182 |
|  |  |  |  |  |  |  |  |  |  |
| **rs2102302** | Controls | % | Cases | % | OR | lower | upper | *P*-value | AIC |
| Codominant |  |  |  |  |  |  |  |  |  |
| A/A | 365 | 42.0 | 353 | 39.6 | 1.00 |  |  | 0.6044 | 2446 |
| A/G | 377 | 43.3 | 400 | 44.9 | 1.10 | 0.90 | 1.34 |  |  |
| G/G | 128 | 14.7 | 138 | 15.5 | 1.11 | 0.84 | 1.48 |  |  |
| Dominant |  |  |  |  |  |  |  |  |  |
| A/A | 365 | 42.0 | 353 | 39.6 | 1.00 |  |  | 0.3187 | 2444 |
| A/G-G/G | 505 | 58.0 | 538 | 60.4 | 1.10 | 0.91 | 1.33 |  |  |
| Recessive |  |  |  |  |  |  |  |  |  |
| A/A-A/G | 742 | 85.3 | 753 | 84.5 | 1.00 |  |  | 0.6495 | 2445 |
| G/G | 128 | 14.7 | 138 | 15.5 | 1.06 | 0.82 | 1.38 |  |  |
| log-Additive |  |  |  |  |  |  |  |  |  |
| 0,1,2 | 870 | 49.4 | 891 | 50.6 | 1.07 | 0.93 | 1.22 | 0.3524 | 2444 |

OR, odds ratio; AIC, Akaike information content.
